# A presença da Geografia de Ptolomeu nas bibliotecas portuguesas: inventário, contextualização e usos de um livro científico antigo

**DOI:** 10.1590/S0104-59702026000100028

**Published:** 2026-08-10

**Authors:** Diego Maguelniski, Fabrício Pedroso Bauab, Francisco Roque de Oliveira

**Affiliations:** i Bolseiro de doutoramento, Universidade Estadual do Oeste do Paraná. Francisco Beltrão – PR – Brasil. diegomag.com@gmail.com; ii Professor e investigador, Universidade Estadual do Oeste do Paraná. Francisco Beltrão – PR – Brasil. fabriciobauab@yahoo.com.br; iii Professor e investigador, Centro de Estudos Geográficos, Instituto de Geografia e Ordenamento do Território/Universidade de Lisboa. Lisboa – Portugal. f.oliveira@edu.ulisboa.pt

**Keywords:** Cláudio Ptolomeu (ca. 90-ca. 170), *Geografia* (livro), Livro científico antigo, História da ciência, Portugal, Claudius Ptolemy (ca. 90-ca. 170, Geography, Ancient scientific book, History of science, Portugal

## Abstract

Neste estudo, procedemos à identificação das edições da *Geografia*, de Cláudio Ptolomeu, impressas até 1730 e existentes nas bibliotecas portuguesas. A maior parte desses exemplares corresponde a edições do século XVI e constitui um indicador muito relevante para a análise dos modos de leitura desse texto científico clássico no contexto do Renascimento português. Para a reconstituição do universo de leitores desse livro, procedemos à identificação das marcas de posse de seus antigos possuidores. Esse conjunto de sinais permite-nos acompanhar a diversidade de usos dessa obra, designadamente aqueles que remetem para os ambientes monásticos e universitários, assim como para os círculos cortesãos e mercantis aos quais o livro mais interessou.

## Abstract (en)

In this study, we have identified the editions of Claudio Ptolemy’s Geography printed up to 1730 and held in Portuguese libraries. Most of these copies correspond to sixteenth-century editions and are a very relevant indicator for analysing the ways in which this classic scientific text was read in the context of the Portuguese Renaissance. In order to reconstitute the universe of readers of this book, we identified the ownership marks of former owners of these books. This set of signs allows us to follow the diversity of uses of this work, in particular those that refer to monastic and university environments, as well as the courtly and mercantile circles that were most interested in this book.

## A redescoberta da Terra segundo Ptolomeu

Redescoberta pelo Ocidente latino no início do século XV, a *Geografia*, de Cláudio Ptolomeu (ca. 90-ca. 170), veio a constituir uma das mais influentes obras do Renascimento europeu, confirmando a estreita ligação que se estabeleceu nessa época entre a geografia antiga e a geografia moderna (Broc, 2019). Último grande representante da ciência grega, o astrónomo, astrólogo, matemático, físico, teórico da música e geógrafo alexandrino Ptolomeu legou três obras de conteúdo muito díspar, mas unidas pelo mesmo propósito de estudarem e representarem o mundo habitado: a *He Mathematiké Syntaxis* (Sintaxe matemática), mais conhecida pelo título árabe *Almagesto*, constitui um tratado de astronomia geocêntrica no qual os diversos componentes do sistema terra-céu são estudados pela geometria e configuram uma conceção harmoniosa e geométrica do universo; o breviário de astrologia *Tetrabiblos*, que apresenta um quadro das influências astrais sobre diversos territórios e que também ocorre com a designação grega *Apotelesmatiká*; e, finalmente, a referida *Geographike Hyphegesis*, *Guia geográfico*, ou simplesmente *Geografia*, obra escrita por volta de 150 e que sistematiza um conjunto de instruções matemático-cartográficas para desenhar tanto um mapa-múndi sobre um plano como mapas regionais, incluindo informação sobre a respetiva topografia (Jacob, 2008; Aujac, 2012).

A *Geografia* de Ptolomeu surge dividida em oito livros, correspondendo a dois grandes tipos de conteúdos: por um lado, elementos teóricos sobre a geografia e a elaboração de mapas do mundo; por outro lado, um catálogo de topónimos, distribuídos por regiões e acrescentados das respetivas coordenadas em latitude e longitude. O primeiro desses oito livros está reservado para a exposição dos princípios cartográficos teóricos, incluindo a “retificação” das dimensões e da imagem do mundo habitado dadas pelo mais recente dos grandes geógrafos que o precederam, Marino de Tiro (ca. 60-ca. 130), que sistematizara o conhecimento resultante do relacionamento de Roma com a China e a costa oriental africana a sul do Equador e de quem só conhecemos aquilo que Ptolomeu escreveu a seu respeito. Nesse primeiro livro da *Geografia*, Ptolomeu inscreve também uma distinção operativa entre geografia e corografia – isto é, as descrições do todo ou de uma parte do globo terrestre –, distinção que seria amplamente glosada pelos geógrafos renascentistas. Por último, apresenta também um conjunto de regras para a projeção do mapa terrestre sobre um suporte plano, de modo a garantir uma visão sinóptica da ecúmena que a observação de um mapa projetado sobre uma secção da esfera não permitia (Aujac, 1998, 2012; Jacob, 2008; Gautier-Dalché, 2009; Broc, 2019).

Os livros II a VII da *Geografia* de Ptolomeu são compostos pelo catálogo de oito mil lugares do mundo conhecido, acrescidos das respetivas posições topográficas, começando na Europa (distribuída em trinta regiões, da Irlanda ao Peloponeso), continuando no continente africano (a Líbia dos Antigos, dividida em oito secções, do atual Marrocos à Etiópia), e terminando nas 44 secções que tratam dos espaços asiáticos, entre a Ásia Menor e a ilha de Taprobana (atual Sri Lanka). Para a última secção do livro VII estão reservadas novas indicações sobre a construção do mapa do mundo e dos mapas regionais, e a descrição da esfera armilar e do próprio mapa-múndi. Por fim, Ptolomeu preenche o livro VIII com a apresentação do seu plano cartográfico, que consistia na realização daquilo que hoje designaríamos como um atlas composto por um mapa geral do mundo em projeção cónica e 26 cartas regionais desenhadas em projeção ortogonal, seguindo esta sequência: dez mapas da Europa, quatro mapas da Líbia-África e 12 mapas da Ásia.

No conjunto, a ecúmena ptolemaica estende-se em largura a 180º (e não a 220º, que era a lição sobre o mundo habitado dada por Marino de Tiro), entre as ilhas Afortunadas (Canárias) e o cabo Catigara, ocupando metade do hemisfério Norte. O limite norte do mundo ficava marcado a 63º N, no paralelo de Tule, enquanto o limite sul era traçado a 16º30’ de latitude sul – e não no trópico de Capricórnio, como Marino fizera. Esse espaço é apresentado como um todo não fragmentado em continentes, desenhando os mares sem comunicação entre si, a exemplo do Índico, que figura como um grande mar interior, constituindo, assim, um dos maiores erros de cálculo da geografia ptolomaica e que o Renascimento europeu se encarregaria de revogar (Jacob, 2008; Aujac, 2012).

O método cartográfico descrito por Ptolomeu esteve na base das 27 cartas que ilustram alguns dos mais antigos manuscritos conhecidos da *Geografia*, elaboradas em finais do século XIII, na circunstância da redescoberta dessa obra pelo erudito bizantino Máximo Planudes (ca. 1255-1305), que a daria a ver ao imperador Andrónico II Paleólogo. Essa redescoberta em Bizâncio colocou termo ao longo eclipse de 12 séculos durante os quais a *Geografia* circulou sobretudo entre eruditos arménios, persas e árabes e antecipou a respetiva transmissão ao Ocidente, via a tradução do grego para latim que Jacopo d’Angelo (ca. 1360-1411) completou por volta de 1406, dedicada ao papa Alexandre V e impressa pela primeira vez em 1475, em Vicenza. Ainda assim, as primeiras edições ocidentais da *Geografia* de Ptolomeu persistiram em apresentá-la sem mapas. Foi apenas quando o cartógrafo alemão Nicolaus Germanus (ativo em Itália entre ca. 1460 e 1477) produziu os mapas que vieram a ilustrar a edição da *Geografia* publicada em 1477, em Bolonha, que se generalizou a prática de associar o texto de Ptolomeu às representações cartográficas da ecúmena que este ensinava a representar (Lourenço, 1999; Berggren, Jones, 2000).

**Figura 1:**
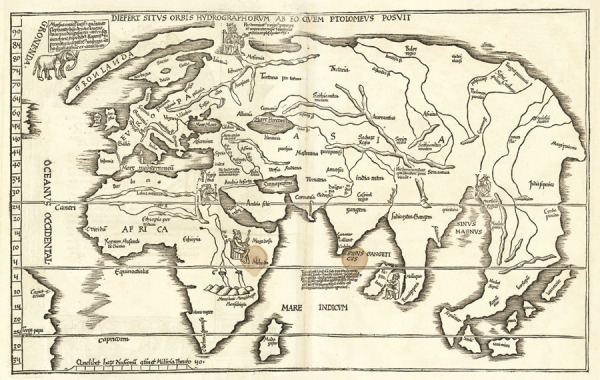
: [*Tabula nova totius orbis*] *Diefert Situs Orbis Hydrographorum ab eo quem Ptolemeus Posuit*. Edição do mapa-múndi de Martin Waldseemüller de 1513 inserto na edição da *Geografia* de Ptolomeu de 1525 (Estrasburgo)

A Itália e a Alemanha – e, em menor medida, a França – constituem os principais polos responsáveis pela difusão da *Geografia* de Ptolomeu na Europa do Renascimento. Das quarenta edições desse livro contabilizadas nos séculos XV e XVI, a maior parte sai de tipografias italianas, seguindo uma sequência cronológica desigual: oito edições entre a edição de Vicenza de 1475 e a de Veneza de 1511, seguidas de um novo conjunto de dez edições concentradas entre 1548 e 1599, todas em Veneza. De permeio, sucedeu o significativo hiato de 17 anos entre as edições romanas de 1490 e 1507, a que não foi certamente alheio o sobressalto geográfico suscitado pelo resultado somado da viagem de Bartolomeu Dias ao Índico e de Cristóvão Colombo às Antilhas e que a incorporação de sucessivas tábuas novas haveria de tentar resolver, a começar por aquelas que passavam a mostrar a intercomunicabilidade entre os oceanos Atlântico e Índico (Figura 1) (Cortesão, 1964). Por seu lado, à parte as edições de Ulm de 1482 e 1484, os prelos alemães de Estrasburgo, Nuremberga, Basileia, Ingolstadt e Colónia são responsáveis pela publicação de 14 edições da *Geografia* de Ptolomeu entre 1513 e 1552, a que acrescerão as edições tardias de Colónia de 1578, 1584 e 1597. Já das oficinas francesas vêm as conhecidas edições de 1535 (Lyon), 1541 (Lyon/Viena) e 1546 (Paris) (Sanz, 1959; Lourenço, 1999; Gautier-Dalché, 2007; Shalev, 2011).

(Fonte: Biblioteca Nacional de Portugal, RES. 1316 A. Obra no domínio público)

Apesar da manifesta escassez de indícios, há muito que se vem fazendo a história da difusão dos princípios fundamentais da *Geografia* de Ptolomeu na Península Ibérica. Os comentários sobre os *Salmos* que o teólogo e hebraísta Jaime Pérez de Valencia (ca. 1408-1490) publicou em Valência em 1484 desenvolvem uma descrição da estrutura da ecúmena alinhada com o princípio ptolemaico que recusa a ideia da intercomunicação dos mares. É possível que Pérez tenha manuseado a cópia manuscrita da *Geografia* existente na Biblioteca da Universidade de Valência e que pertenceu ao rei Fernando de Aragão, duque da Calábria (1488-1550) (Navarro Brotons, 1983). No ano seguinte ao da publicação de *Commentaria in Psalmos* de Pérez, o conhecimento do conteúdo da *Geografia* de Ptolomeu é também atestado em Portugal via a *Oração de obediência* do rei dom João II, pronunciada pelo jurisconsulto e diplomata Vasco Fernandes de Lucena (?-1499?) diante do papa Inocêncio VIII, em Roma, celebrando as navegações portuguesas até à proximidade do Promontório Prasso. Igualmente, parece razoável supor que um ou vários manuscritos da *Geografia* tenham sido manuseados pelo círculo erudito do infante dom Henrique (1394-1460), no contexto da preparação das viagens de navegação ao longo da costa ocidental africana (Randles, 1995, 2000).

Quanto aos princípios da geografia matemática ptolemaica – ou, pelo menos, aqueles de Marino de Tiro recuperados pelo texto de Ptolomeu –, encontramo-los plasmados na pequena obra *Isagogicon cosmographiae*, que o humanista Antonio de Nebrija (1444-1522) redigiu em Salamanca entre 1487 e 1490 e que seria publicada na mesma cidade entre 1497 e 1505, sob o título *In cosmographiae libros introductorium*. Em contrapartida, o conceito de globo terrestre de superfície esférica contínua, coberto por uma malha de meridianos e paralelos, como ensinado pelo método de Marino e Ptolomeu – e entretanto validado pela experiência das navegações portuguesas, que tinham demonstrado a habitabilidade da zona tórrida e a existência de antípodas –, está no cerne do *Physices compendium* (1520) de Pedro Margalho (ca. 1473-1556), jurista, cosmógrafo e teólogo português, professor na Universidade de Salamanca nesses anos em que esta sua obra foi publicada (Randles, 1995; Gautier-Dalché, 2007, 2009; Portuondo, 2013). A tradução, seguida de comentário, do primeiro livro da *Geografia* de Ptolomeu que o matemático Pedro Nunes incorporou no seu *Tratado da esfera* (1537) confirmou o lugar dessa obra do geógrafo alexandrino entre a principal bibliografia geográfica e cartográfica que circulou em Portugal no século XVI, assim como o papel que desempenhou na definição da nova imagem do mundo e do seu conceito teórico e matemático.

## Opções metodológicas

Há cerca de um século, o historiador da cartografia Armando Cortesão empreendeu uma primeira contagem dos exemplares da *Geografia* de Ptolomeu à guarda da atual Biblioteca Nacional de Portugal (BNP). Os resultados desse inquérito aparecem numa extensa nota de rodapé nas páginas iniciais do primeiro volume de *Cartografia e cartógrafos portugueses dos séculos XV e XVI*. Cortesão (1935) dá conta da presença nesse espólio das edições da *Geografia* de 1513 (Estrasburgo), 1520 (Estrasburgo), 1524 (presumivelmente, a edição de 1525 de Estrasburgo) e 1730 (Amesterdão), para além de um exemplar datado de 1619 da edição de Pieter van den Berghe, que deverá corresponder àquela publicada por Pieter de Bert em 1618-1619, em Leyden, e que inclui 28 cartas reconstituídas por Gerard Mercator que vinham da edição da *Geografia* de Ptolomeu de 1578 (Colónia), além de 14 novos mapas provenientes de diversas edições anteriores do *Atlas* de Joost de Hondt (Cortesão, 1935; Gautier-Dalché, 2007).

Mais recentemente, Fernanda Guedes de Campos (2013) procedeu ao levantamento de exemplares dessa obra de Ptolomeu na mesma BNP. Fê-lo no âmbito da pesquisa de doutoramento que realizou sobre as bibliotecas das casas religiosas de Lisboa, no século XVIII, razão pela qual se restringiu àqueles livros que evidenciavam marcas de posse. Nesse trabalho, foram identificados seis títulos, dos quais apenas cinco correspondem, de facto, a edições da *Geografia*: as edições de Basileia de 1533, 1540 (na verdade, 1542) e 1545, a edição de Lyon/Viena de 1541 e a edição de Veneza de 1561. À parte esse conjunto de livros, Campos (2013) também dá conta da existência de um exemplar da *Opera omnia* de Ptolomeu impressa em Basileia, em 1541, por Heinrich Petri*.* Publicada com o título *Claudii Ptolemaei Pelusiensis Alexandrini omnia quae extant opera, Geographia excerpta*, trata-se de uma coletânea de traduções e comentários de alguns dos principais textos de astronomia e astrologia de Ptolomeu – o *Almagesto* e o *Tetrabiblos*, designadamente –, ou atribuídos a Ptolomeu – como é o caso do parapegma astrometeorológico *Inerrantium stellarum significationes* –, mas que não inclui qualquer trecho da *Geografia* (Juste, 2022, 2024).

Até hoje, o mais extenso inquérito a esses objetos foi realizado por João Daniel Lourenço, quando publicou na revista *Evphorosyne*, em 1999, uma importante resenha sobre a receção da *Geografia* de Ptolomeu no Portugal do Renascimento. Nesse estudo, Lourenço identificou 51 exemplares dessa obra nas bibliotecas públicas portuguesas, sendo dois quatrocentistas e 49 do século XVI. Como então sublinhou esse autor, boa parte desses livros é proveniente de bibliotecas monásticas, que conservaram durante séculos a qualidade de principais centros depositários do conhecimento livresco. É o caso de três dos exemplares da *Geografia* guardados na BNP que haviam pertencido à livraria do Convento de São Francisco de Xabregas, em Lisboa, que fora sede da Província dos Algarves da Ordem dos Frades Menores da Observância: as anteriormente referidas edições de Basileia de 1533 e 1542, em latim, e a edição de Veneza de 1561, em italiano. No seu estudo, Lourenço (1999) confirmou também para Portugal a geografia europeia das edições da *Geografia* de Ptolomeu que sinalizámos, contabilizando 21 exemplares italianos, 23 alemães e apenas sete exemplares de edições francesas.

Propomo-nos retomar os exercícios executados por Armando Cortesão, João Lourenço e Fernanda Campos, mas começando por alargar o intervalo cronológico até 1650. A definição desse primeiro limite está alinhada com a análise sobre os modos de leitura, compreensão e interpretação da *Geografia* de Ptolomeu durante o Renascimento apresentada por Patrick Gautier-Dalché (2007), e que remete a questão para os termos próprios da história cultural. Atenta também nas observações de Leslay Cormack (2011) sobre a persistência da autoridade de Ptolomeu até meados do século XVII nos contextos geográfico-matemáticos ingleses. Ainda assim, registámos também os (poucos) exemplares desse livro publicados até 1730 e existentes nas bibliotecas públicas portuguesas, de modo a possibilitar o simultâneo cruzamento de dados com dois dos inventários de cartografia antiga que são recorrentemente mobilizados para o estudo dessas temáticas: *Antique maps*, de Carl Moreland e David Bannister (1995), e *Ptolemy’s Geography: a brief account of all the printed editions down to 1730*, de Henry N. Stevens (1908), título do catálogo específico que este autor produziu a propósito da coleção legada por Henry Stevens, seu pai, à Biblioteca de Edward E. Ayer, de Chicago (atual Newberry Library). Este último catálogo era explicitamente devedor da bibliografia da *Geografia* de Ptolomeu que o bibliógrafo e bibliotecário norte-americano Wilberforce Eames publicara em 1886, em Nova York, com o título de *A list of the editions of Ptolemy’s Geography, 1475-1730* (Eames, 1886; Stevens, 1908).

Tal como acabámos por aplicar um duplo critério para a definição do intervalo cronológico abrangido pela nossa análise, cruzámos também uma dupla metodologia para o rastreio dos exemplares da *Geografia* de Ptolomeu à guarda de bibliotecas portuguesas. A pesquisa inicial foi realizada a partir da Base Nacional de Dados Bibliográficos e da Biblioteca Comum (Catálogo Coletivo das Bibliotecas das Instituições de Investigação e Ensino Superior de Portugal), que completámos com os seguintes catálogos de referência: catálogos das Bibliotecas da Universidade de Lisboa, Universidade de Coimbra, Universidade do Porto e Universidade Católica Portuguesa; catálogos das Bibliotecas de Lisboa, Bibliotecas Municipais do Porto, Biblioteca Pública de Évora e, ainda, da Biblioteca da Academia das Ciências de Lisboa e da Rede de Bibliotecas, Museus e Monumentos de Portugal.

O segundo passo correspondeu ao confronto das ocorrências verificadas nos catálogos bibliográficos com os exemplares existentes nas diversas bibliotecas sinalizadas, sendo que a maior parte dos títulos identificados se encontra sob guarda da BNP. Também realizámos consulta direta em duas instituições previamente escolhidas, mas cujos catálogos não se encontram disponíveis em linha: Biblioteca do Palácio Nacional de Mafra e Sociedade de Geografia de Lisboa. Apesar da natureza privada deste último organismo cultural, científico e histórico, considerou-se que os vínculos que mantém desde a sua fundação, em 1875, com o património bibliográfico das principais bibliotecas do Estado português justificavam uma pesquisa alargada ao respetivo espólio. Manuseámos ainda os principais catálogos de livro antigo disponíveis para algumas coleções portuguesas, tanto relativos a incunábulos, como a impressos – designadamente aqueles dedicados ao livro científico antigo (Mendes, 1988; Leitão, Martins, 2004; Cristóvão, 2008). Finalmente, procedemos a consultas complementares no catálogo da Biblioteca Nacional do Rio de Janeiro, para assinalar algumas correspondências com os catálogos portugueses, como adiante se explicará.

Para cada exemplar confirmado da *Geografia* de Ptolomeu identificado no decurso deste levantamento, verificámos o título completo, idioma, data, impressor, local de edição, cota de catálogo e marcas de posse de antigos possuidores. Também neste aspeto, tivemos por base os referidos exercícios realizados por João Lourenço (1999) e Fernanda Campos (2013), ao se debruçarem sobre as marcas de leitura encontradas em alguns dos exemplares da obra datados do século XVI e que constituem matéria específica da receção do texto antigo e das práticas de leitura. Deliberadamente, não estendemos a pesquisa aos conteúdos de bibliotecas desaparecidas e entretanto reconstituídas a partir de evidências lacunares, como é o caso da livraria do duque Teodósio I de Bragança (1510?-1563), mesmo quando sabemos que incorporavam exemplares da *Geografia* de Ptolomeu, como foi o caso dessa notável biblioteca renascentista (Buescu, 2016).

## Nova contagem da *Geografia* de Ptolomeu em Portugal

Identificámos no acervo das diversas bibliotecas portuguesas que pesquisámos um conjunto de 63 exemplares da *Geografia* de Ptolomeu editados até 1730 (Quadro 1). Destes, três exemplares correspondem a incunábulos, 55 exemplares a livros editados no decorrer do século XVI, restando cinco exemplares publicados entre o início do século XVII e 1730. Ao conjunto podemos acrescentar a identificação de seis exemplares do *Tratado da esfera* de Pedro Nunes, impressos em Lisboa em 1537 pelo tipógrafo de origem francesa Germain Gaillard, e que incluem a referida tradução comentada do primeiro livro da *Geografia* de Ptolomeu. Localizámos ainda uma primeira edição da *Historiae Britanicae, Saxonicae, Anglo-Danicae scriptores XV*, de Thomas Gale (1635?-1702), que inclui extratos da parte inicial do livro VIII da *Geografia* correspondentes às Ilhas Britânicas (publicado em Oxford, em 1691). Tal como no caso do tratado de Pedro Nunes, também a coletânea de Thomas Gale não corresponde, em rigor, a uma edição da *Geografia* de Ptolomeu, razão pela qual desagregámos qualquer dessas ocorrências da contabilidade reservada aos exemplares propriamente ditos da *Geografia*.

**Quadro 1:** : Distribuição de exemplares da *Geografia* de Ptolomeu pelas bibliotecas portuguesas, edições até 1730

Data	Local de edição e impressores	Localização dos espécimes
1486	Ulm, Johannes Rerger	BGUC
1490	Roma, Pietro de la Torre	BA, BNP
1507	Roma, Bernardino di Vidali	BPMP
1508	Roma, Bernardino di Vidali	ACL, BNP
1511	Veneza, Jacopo Pencio de Leuco	BNP
1513	Estrasburgo, Johannes Schott	BGUC, BNP
1514	Nuremberga, Johannes Struchs	ACL, BNP
1520	Estrasburgo, Johannes Schott	BNP, FLUL
1525	Estrasburgo, Johann Grüninger e Johannes Koberger	BGUC, BNP, MUHNAC
1533	Basileia, Hieronymus Froben [e Nikolaus Episcopius?]	BNP
1533	Ingolstadt, Peter Schöffer	BA, BGUC
1535	Lyon, Melchieor Trechsel e Gaspard Trechsel	BGUC, BPMP, FAFCUP, MUHNAC
1540	Colónia, Johann von Remund	BNP
1541	Viena, Gaspard Trechsel	BNP, BPMP
1542	Basileia, Heinrich Petri	BNP
1545	Basileia, Heinrich Petri	ACL, BNP, BPMP
1546	Paris, Chrétien Wechel	BNP
1548	Veneza, Nicòlo Bascarini	BJUC, BPMP
1552	Basileia, Heinrich Petri	BGUC, BNP
1561	Veneza, Vincenzo Valgrisi	BCM, BNP
1562	Veneza, Vincenzo Valgrisi	BJUC, BNP, BPE
1574	Veneza, Giordano Ziletti	BGUC, BNP, BPMP, PNM, SGL
1584	Colónia, Gottfried von Kempen	MUHNAC
1597	Colónia, Peter Keschedt	BPMP
1598	Veneza, Giouanni Battista Calignani e Giorgio Calignani	BCM, BNP, BUJPII
1598	Veneza, Herdeiros de Melchior Sessa	BNP
1605	Frankfurt/Amesterdão, Jan Theunisz.? para Jodocus Hondius e Cornelius Nicolai	BNP, BPMP
1621	Pádua, Paolo Galignani e Francesco Galignani	BUJPII
1730	Amesterdão, Rudolf Wetstein, Jacob Wetstein e William Smith	BMUC, BNP
**Siglas:** ACL - Academia das Ciências de Lisboa BA - Biblioteca da Ajuda, Lisboa BCM - Biblioteca Central de Marinha, Lisboa BGUC - Biblioteca Geral da Universidade de Coimbra BJUC - Biblioteca Joanina da Universidade de Coimbra BMUC - Biblioteca Matemática da Universidade de Coimbra BNP - Biblioteca Nacional de Portugal BPE - Biblioteca Pública de Évora BPMP- Biblioteca Pública Municipal do Porto BUJPII - Biblioteca Universitária João Paulo II, Universidade Católica Portuguesa, Lisboa FAFCUP - Fundo Antigo da Faculdade de Ciências da Universidade do Porto FLUL - Faculdade de Letras da Universidade de Lisboa MUHNAC - Museu Nacional de História Natural e da Ciência, Universidade de Lisboa PNM - Palácio Nacional de Mafra SGL - Sociedade de Geografia de Lisboa		

Fonte: Elaborado pelos autores.

Os três incunábulos localizados correspondem a uma edição alemã e a outra italiana, ambas em latim, como seria norma nas edições da *Geografia* de Ptolomeu até meados do século XVI. Trata-se da edição de Ulm devida a Johannes Reger, de 1486, estante na Biblioteca Geral da Universidade de Coimbra, e de dois exemplares da edição romana de Pietro de la Torre, de 1490 – uma localizada na BNP e a outra na Biblioteca da Ajuda, em Lisboa, cujo acervo principal tem origem na antiga Biblioteca Real portuguesa.

Confirmando o que sabemos sobre o continuado interesse dos prelos europeus pela *Geografia* de Ptolomeu até ao final da década de 1540, também a maioria das edições dessa obra que identificámos foi impressa na primeira metade desse século: em concreto, 36 exemplares publicados entre a edição de Roma de 1507 e a edição de Veneza de 1548, contra os 19 exemplares contados entre a edição de Basileia de 1552 e os quatro exemplares de edições venezianas de 1598. Para o século XVII, foram localizados apenas dois exemplares da edição de Frankfurt/Amesterdão de 1605 e um exemplar de edição impressa em Pádua, em 1621. Dois exemplares da edição impressa em Amesterdão, em 1730, conferem com as únicas edições da *Geografia* de Ptolomeu do século XVIII encontradas até a esse mesmo ano, que ficou definido como limite cronológico para o nosso inquérito.

Também a distribuição relativa das existências por origem geográfica das edições traduz o panorama mais geral da oferta deste título pelas diversas tipografias italianas, alemãs e francesas que o disponibilizaram no mercado. A respeito das edições italianas da *Geografia* incorporadas nessas bibliotecas, temos que oito exemplares do século XVI correspondem a edições datadas de entre 1500 e 1540, havendo 16 exemplares da segunda metade do mesmo século. Quanto às edições alemãs – cujo ritmo de edição na Europa foi mais uniforme ao longo do século XVI, ainda que particularmente acelerado entre 1520 e 1540 (Lourenço, 1999) –, verificámos que 22 das edições quinhentistas localizadas foram publicadas até ao final da década de 1540, contra apenas quatro exemplares da segunda metade da mesma centúria. Por último, localizámos exemplares de todas as edições de tipografia francesa, assim distribuídos: quatro da edição de Lyon de 1535, três da edição de Lyon/Viena de 1541 e um exemplar da edição de Paris de 1546. A propósito, diga-se que apenas não localizámos nas referidas bibliotecas quaisquer exemplares de sete das 31 edições da *Geografia* de Ptolomeu do século XVI: as edições alemãs de 1522 (Estrasburgo), 1540 (Basileia), 1541 (Basileia) e 1578 (Duisburgo), assim como estão em falta exemplares das duas edições venezianas de 1564 (Moreland, Bannister, 1995; Gautier-Dalché, 2007).

As lacunas nos catálogos portugueses atuais só são realmente expressivas para os incunábulos e para as edições de tipografia alemã e neerlandesa do século XVII e início do século XVIII. Acresce que todo esse espólio deverá ter pontos de contato com alguns dos exemplares quinhentistas e seiscentistas da *Geografia* de Ptolomeu incorporados na Biblioteca Nacional do Rio de Janeiro, em particular aqueles provenientes da Real Biblioteca e do Acervo Antigo, que têm a sua génese na transferência para o Brasil dos acervos da Real Biblioteca da Ajuda e da Livraria da Casa do Infantado, entre 1810 e 1811, e na subsequente fundação da Real Biblioteca Pública da Corte do Rio de Janeiro, em 1814 (Schwarcz, 2002; Rodrigues, 2024). Tal é o caso dos exemplares das edições de 1533 (Basileia), 1535 (Lyon), 1561 (Veneza) e 1605 (Frankfurt/Amesterdão).

O mesmo se poderá aplicar ao exemplar da primeira edição do *Tratado da esfera*, de Pedro Nunes, existente na Biblioteca Nacional do Rio de Janeiro, que ostenta sucessivas marcas de posse do bibliófilo Diogo Barbosa Machado (1682-1772), do diplomata António de Araújo de Azevedo, conde da Barca (1754-1817), e da Real Biblioteca. Já o exemplar da edição da *Geografia* da edição de Ulm de 1486, também presente no espólio dessa biblioteca, sabe-se provir da coleção da imperatriz dona Teresa Cristina Maria (1822-1889), tendo sido doado pelo imperador dom Pedro II à Biblioteca Nacional brasileira, o que comprova uma procedência distinta daquela dos atuais catálogos públicos portugueses, muito provavelmente, napolitana (Barreto, 2009).

## A posse dos livros

Completamos a nossa leitura cruzando algumas indicações sobre a distribuição dos exemplares da *Geografia* de Ptolomeu que localizámos nas principais bibliotecas de Portugal com a descrição e a contextualização, necessariamente sumárias, das marcas de posse apostas em muitos desses livros. A relevância dos testemunhos de propriedade inscritos nos livros tem sido sobejamente atestada para os estudos na área da história do livro e da leitura (Pearson, 1988; Navarro Bonilla, 2003; Martín Abad, 2004; Campos, 2013, 2015; Villalta, 2015). Vale também notar que a ausência de marca institucional em muitos dos espécimes que analisámos não invalida uma proveniência comum, sobretudo quando os livros em causa integravam originalmente espólios de casas religiosas, para algumas das quais se intui que a prática de não inscrever qualquer sinal de propriedade nesses objetos ocorria de forma sistemática. Esse parece ter sido o caso da grande Biblioteca do Convento de São Francisco da Cidade, sede da Província de Portugal da Ordem dos Franciscanos Observantes, que teve 25 mil volumes arrolados em 1834, dois anos antes de terem sido integrados na Real Biblioteca Pública (Campos, 2013, 2015).

Passando à contagem global das existências, a primeira evidência decorre do elevado número de exemplares da *Geografia* de Ptolomeu que se encontram na BNP, em Lisboa – 28 títulos, de um universo de 63 títulos, como referimos. Essa circunstância decorre sobretudo do conhecido processo de constituição desse acervo a partir dos fundos bibliográficos das ordens religiosas extintas em 1834 pelo liberalismo. Antes disso, durante o consulado (1750-1777) de Sebastião José de Carvalho e Melo, marquês de Pombal, o património bibliográfico existente nos colégios e casas da Companhia de Jesus – extinta em Portugal em 1759 – passou para a posse da Real Mesa Censória, a instituição antecessora da Real Biblioteca Pública da Corte, por sua vez, a mais antiga antecessora formal da atual BNP (Domingos, 1995; Barata, 2003; Cabral, 2014a).

Encontramos na BNP exemplares da *Geografia* ptolomaica distribuídos pela totalidade do intervalo cronológico abrangido pelo nosso inquérito – da mencionada edição de Roma de 1490 à também referida edição de Amesterdão de 1730. Com exceção dos anos de 1580 – quando apenas ocorre a edição publicada em Colónia, em 1584 –, localizamos na instituição exemplares publicados em todas as restantes décadas entre a última do século XV e a primeira do século XVII: além da edição de 1490, que acabámos de referir, também exemplares das edições de 1508 (Roma), 1511 (Veneza), 1513 (Estrasburgo), 1514 (Nuremberga), 1520 (Estrasburgo), 1525 (Estrasburgo), 1533 (Basileia), 1540 (Colónia), 1541 (Viena), 1542 (Basileia), 1545 (Basileia), 1546 (Paris), 1552 (Basileia), 1561 (Veneza), 1562 (Veneza), 1574 (Veneza), 1598 (Veneza) e 1605 (Frankfurt/Amesterdão).

**Figura 2:**
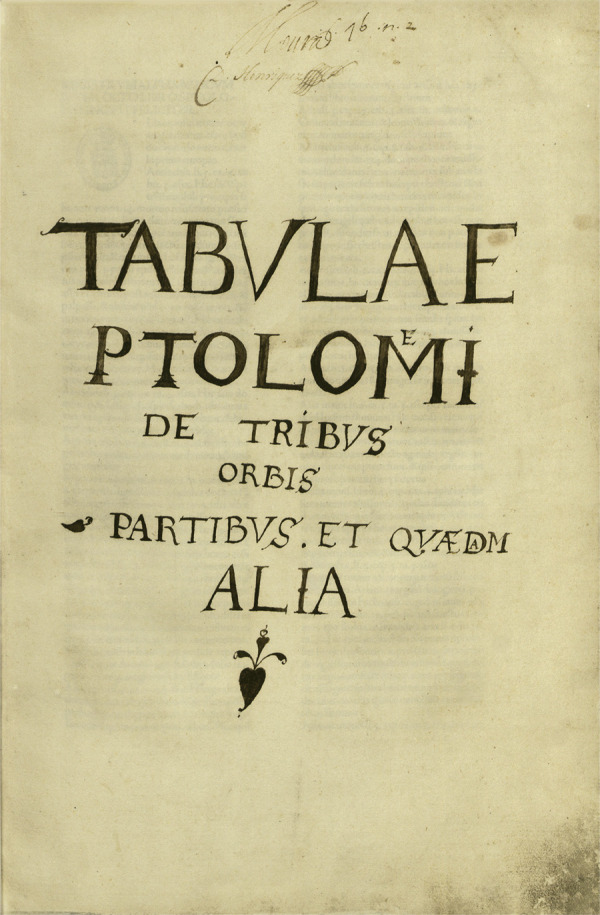
: Folha de rosto da edição da *Geografia* de Ptolomeu de 1525 (Estrasburgo)

Como começámos por dizer a partir da pista dada por Lourenço (1999), encontramos na BNP exemplares correspondentes a três edições da *Geografia* – 1533, 1542 e 1561 – provenientes da livraria do Convento de São Francisco de Xabregas, de Lisboa. Deverá também ter pertencido a essa biblioteca um exemplar da edição de Estrasburgo de 1525 que tem a seguinte marca de posse: “à Comunidade da Beata Maria de grã(?) Lisboa”. Por seu turno, o incunábulo romano de 1490 pertenceu à livraria da Congregação do Oratório, mas ostenta uma segunda marca de posse de António Henriques da Silveira (1725-1811), conselheiro real, lente da Primeira Cadeira Analítica de Direito Canónico da Universidade de Coimbra, desembargador do Paço e figura relevante do iluminismo português (Fonseca, 2003) (Figura 2).

(Fonte: Biblioteca Nacional de Portugal, INC. 494. Obra no domínio público)

**Figura 3:**
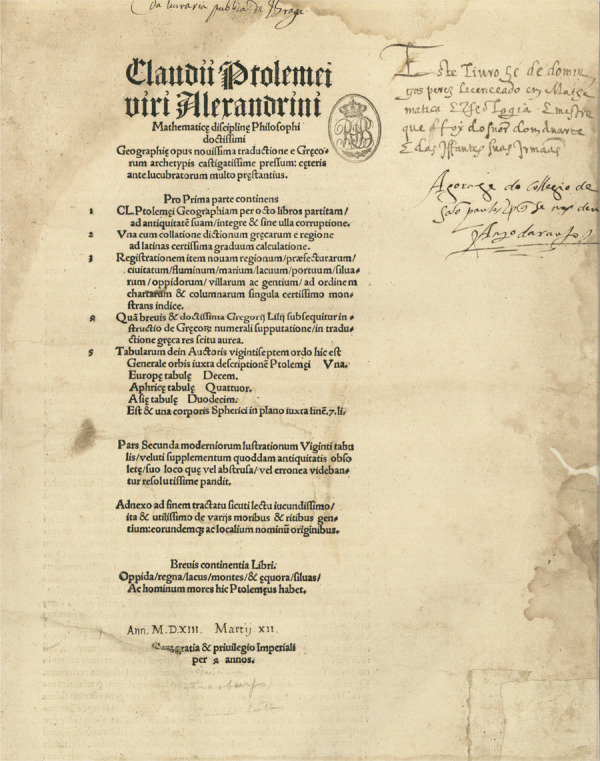
: Folha de rosto da edição da *Geografia* de Ptolomeu de 1513 (Estrasburgo)

Numa anotação manuscrita no fólio 1 do exemplar da edição de Estrasburgo de 1513, indica-se que “Este livro é de Domingos Peres licenciado em Matemática e Teologia e mestre que foi do Senhor Dom Duarte e das infantas suas irmãs. / Agora é do colégio de São Paulo porque nos deu António de Araújo” (Figura 3). A partir do estudo de Lourenço (1999), sabíamos já que este Domingos Peres possuía ainda exemplares do *Almagesto* (publicado em Veneza, em 1515) e dos *Opera varia* (em Nuremberga, em 1535), de Ptolomeu, além de um exemplar do *De radio astronomico et geometrico liber*, de Gemma Frisius, todos eles posteriormente integrados na livraria do Colégio jesuíta de São Paulo de Braga, como também veio a acontecer com esse exemplar da *Geografia*. Encontramos também sinais de transmissão sucessiva de posse num dos exemplares da edição de 1533, que tanto pertenceu ao latinista padre Diogo Mendes de Vasconcelos (1523-1599) – sucessivamente embaixador régio ao Concílio de Trento, cónego teólogo de Évora e inquisidor da Fé nesta mesma cidade do sul de Portugal (Freire, 1964) –, como integrou a livraria do Convento de Santa Maria de Scala Coeli, em Évora, da Ordem da Cartuxa. Essa livraria, mandada encerrar em 1834, encontra-se hoje dispersa por várias instituições públicas portuguesas – Arquivo Nacional Torre do Tombo, Biblioteca Central de Marinha, Biblioteca Pública de Évora e Arquivo Distrital de Évora, para além da própria BNP (Mendes, 2016).

(Fonte: Biblioteca Nacional de Portugal, CA. 152 V. Obra no domínio público)

**Figura 4:**
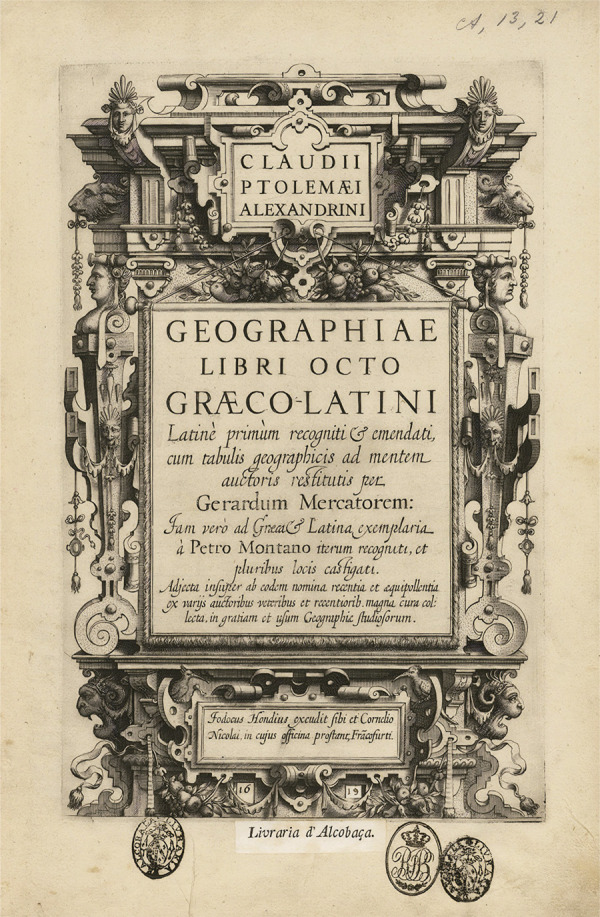
: Folha de rosto da edição da *Geografia* de Ptolomeu de 1562 (Veneza)

Um exemplar da edição de Colónia de 1540 indicia posse pretérita “Do P[adre] João Ba[ptista] de Castro”, referência correspondente ao Padre João Baptista de Castro (1700-1775), presbítero secular vinculado à Patriarcal de Lisboa, que foi autor de obras como o *Roteiro terrestre de Portugal* (1748) e o *Mapa de Portugal antigo e moderno* (1762), onde ocorrem múltiplas citações de Ptolomeu (Curado, 2024). Por sua parte, um exemplar da edição vienense de 1541 traz marca de pertença à biblioteca do mosteiro de São Vicente de Fora, da Ordem dos Cónegos Regrantes de Santo Agostinho, enquanto o de 1545 pertenceu ao Convento de Nossa Senhora da Conceição do Monte Olivete, da Ordem dos Ermitas Descalços de Santo Agostinho. Já a marca de posse da livraria do Convento de Nossa Senhora da Graça, da Ordem dos Ermitas de Santo Agostinho, aparece em exemplares da *Geografia* de 1552 e 1598 – a mesma livraria a que pertenceu o exemplar da *Opera omnia* de Ptolomeu, de 1541, atrás referido (Campos, 2013). Proveniente do colégio jesuíta do Espírito Santo, em Évora, a BNP guarda o exemplar da edição da *Geografia* publicada em 1562, em Veneza. A edição de Frankfurt/Amesterdão, de 1605, que combina a edição latina de Pieter van den Berghe com a versão grega desta mesma obra de Ptolomeu editada por Mercator, traz o *ex-libris* da “Livraria d’Alcobaça” na base da folha de rosto (Figura 4). É o único dos espécimes listados que indicia essa proveniência do importante mosteiro cisterciense de Santa Maria de Alcobaça, cujos livros, mapas e estampas foram enviados para Lisboa entre 1834 e 1838, para darem entrada na Biblioteca Pública (Barata, 2003; Ormeling, 2016).

(Fonte: Biblioteca Nacional de Portugal, CA. 156 V. Obra no domínio público)

Dissonante desse conjunto de marcas de posse associadas a um contexto essencialmente religioso, está o exemplar de 1598 saído da prensa veneziana dos irmãos Calignani, que pertenceu a Nicolò Maria Micone. Próspero mercador genovês que residiu em Lisboa entre as décadas de 1620 e 1670, Micone esteve envolvido no comércio das especiarias da Ásia, dos tecidos italianos e do açúcar brasileiro, tendo sido um dos três acionistas fundadores da Companhia Geral do Comércio do Brasil, instituída em 1649 (Alessandrini, 2009, 2015). Uma segunda marca de posse atesta que este mesmo exemplar passou depois para as mãos de José da Silva Pessanha (1717-1775), sucessivamente embaixador de Portugal em Nápoles e Madrid durante o governo do marquês de Pombal, e que veio a constituir uma notável biblioteca particular de livros antigos (Domingos, 1998). Por sua vez, o bibliófilo António Alberto Marinho Duarte de Sousa (1896-1950) acabou por ser o dono de um exemplar da edição de 1561, que seria integrado na Biblioteca Nacional na década de 1950 (Portugal, 1974).

## Para além da Biblioteca Nacional

O segundo conjunto mais numeroso de exemplares das diversas edições quinhentistas e seiscentistas da *Geografia* de Ptolomeu pertença das bibliotecas portuguesas que fixámos encontra-se à guarda da Biblioteca Pública Municipal do Porto. Tal como em Lisboa, também se determinou que os fundos dos conventos extintos do distrito do Porto e regiões limítrofes, assim como as livrarias sequestradas aos partidários do regime deposto do rei dom Miguel (1828-1834), dessem entrada na Real Biblioteca Pública do Porto, instituição criada em 1833 para exercer a tutela sobre esses bens. Essa incorporação de livros e arquivos na esfera pública estender-se-ia a conventos extintos em territórios mais afastados, como foi o caso do mosteiro beneditino de Tibães, em Braga, e do mosteiro de Santa Cruz de Coimbra, dos Cónegos Regrantes de Santo Agostinho, perfazendo um conjunto de 25 bibliotecas religiosas cujos fundos foram então transferidos pelo Governo liberal para a nova grande biblioteca da cidade do Porto (Barata, 2003, 2005; Cabral, 2014a).

Cinco dos oito exemplares da *Geografia* de Ptolomeu rastreados na Biblioteca Pública Municipal do Porto datam da primeira metade do século XVI, correspondendo às edições em latim de 1507 (Roma), 1535 (Lyon), 1541 (Lyon/Viena) e 1545 (Basileia), e, ainda, à edição em italiano de 1548 (Veneza). Os restantes exemplares conferem com a edição veneziana de 1574 e com as edições alemãs de 1597 (Colónia) e 1605 (Frankfurt/Amesterdão). Desses exemplares, os quatro mais antigos têm inscritas as marcas de posse originais da “Livraria de Sta. Cruz de Coimbra”, enquanto o exemplar de 1574 tem marca que informa ter servido aos frades do mosteiro de São João do Porto, da Ordem dos Pregadores (dominicanos).

Fora os dois exemplares desse conjunto que não revelam marcas de posse – 1548 e 1605 –, aquele de 1597 assinala ter pertencido a “Henricus Ghijm, ilustre erudito”. Acresce que o Fundo Antigo da Faculdade de Ciências da Universidade do Porto custodia um exemplar da edição da *Geografia* de Ptolomeu de Lyon, de 1535, em cuja folha de rosto surge uma primeira marca de posse parcialmente indecifrável que diz “Da Livraria do Colégio de São [?]”. Esta é seguida dos carimbos que indicam “Real Academia do Porto” e “Biblioteca da Academia Politécnica do Porto” – correspondendo, respetivamente, à Academia Real de Marinha e Comércio (1803-1837) e à escola de ensino superior técnico criada em 1837 para substituir essa academia, e que estaria na origem da Escola de Engenharia da Faculdade de Ciências da Universidade do Porto, fundada em 1911.

No conjunto, a Biblioteca Joanina da Universidade de Coimbra, a Biblioteca Geral e a Biblioteca Matemática da mesma universidade custodiam dez dos 63 exemplares da *Geografia* de Ptolomeu que contámos e cujas edições podem-se conferir no Quadro 1. As origens da Biblioteca Joanina remontam ao início do século XVI, mas ganhou projeção a partir de 1716, durante o reinado de dom João V (1707-1750) (Cabral, 2014b). O núcleo de Reservados da Biblioteca Geral da Universidade de Coimbra esteve originalmente instalado em duas dependências anexas à Biblioteca Joanina, até à sua transferência para o novo edifício, em 1957-1958, o que indicia que os dois espólios coabitaram por longo tempo no mesmo espaço (Universidade de Coimbra, 1970). Já o fundo especial da Biblioteca Matemática, designado Livraria de Luciano Pereira da Silva (1864-1926), reúne o valioso núcleo bibliográfico constituído sobretudo por obras de história da náutica e cartografia antiga compiladas por esse destacado historiador da ciência, as quais foram incorporadas na Biblioteca Matemática, em 1929. Apesar da ausência de marcas de pertença que o atestem definitivamente, parte desse espólio parece ter pertencido ao Observatório Astronómico da Universidade de Coimbra – como é o caso de um exemplar do *Almagesto* de Ptolomeu impresso em 1528, em Veneza. Por seu turno, pelo menos alguns dos livros que foram do Observatório Astronómico tinham pertencido à livraria do Mosteiro de Santa Cruz de Coimbra (Biblioteca Matemática, 2014; Tenreiro, 2022).

Ainda durante a regência de dom Pedro IV, em 1832, foi suspenso o envio de livros dos conventos extintos de Coimbra para o Porto, sendo determinado que fossem incorporados na Universidade de Coimbra (Barata, 2003; Amaral, 2014). O processo de arrecadação que se seguiu foi lento e atribulado, mas ainda assim restam vestígios dele na maioria dos exemplares da *Geografia* da Biblioteca Geral que localizámos: marca de posse da livraria do Colégio Real de São Paulo de Coimbra, da Ordem dos Ermitas de São Paulo, no exemplar de 1525, de Estrasburgo; marca de posse da livraria do Colégio dos Cónegos Regulares de Santo Agostinho no exemplar de 1535, de Ingolstadt; marca de posse de Santa Cruz de Coimbra no exemplar de 1574, de Veneza; e marca de posse do Colégio de Nossa Senhora do Carmo de Coimbra no exemplar de 1552, de Basileia. Diga-se, também, que uma segunda marca de posse no exemplar de 1535 atesta que o livro transitou posteriormente para a Biblioteca do Liceu Central de José Falcão, que constituiu uma secção da Universidade de Coimbra entre 1836 e 1880, sucedendo ao extinto Colégio das Artes.

Em sete das bibliotecas de Lisboa e da sua região encontrámos 15 dos restantes 16 exemplares da *Geografia* de Ptolomeu contabilizados neste estudo. Os títulos relativos a três edições – 1525 (Estrasburgo), 1535 (Lyon) e 1584 (Colónia) – estão à guarda do Museu Nacional de História Natural e da Ciência, organismo da Universidade de Lisboa que ocupa o edifício onde funcionaram o Noviciado da Cotovia da Companhia de Jesus (1619-1759), o Real Colégio dos Nobres (1761-1837) e a Escola Politécnica (1837-1911), a qual deu lugar à Faculdade de Ciências da Universidade de Lisboa (Veiga, 2009; Giugevich, Leitão, 2012). As respetivas marcas de posse confirmam a proveniência dos fundos antigos da Companhia de Jesus. Por seu turno, a biblioteca da Academia das Ciências de Lisboa integra exemplares de 1508 (Roma), 1514 (Nuremberga) e 1545 (Basileia), todos com marca de posse da Livraria do Convento de Nossa Senhora de Jesus da Ordem Terceira de São Francisco.

Em vez do que aconteceu com a generalidade das antigas livrarias conventuais, a maior parte da vasta biblioteca do Convento de Jesus não transitou para o Depósito de Livros dos Extintos Conventos, tendo permanecido *in situ*, uma vez que, em 1738, foi entregue à Academia das Ciências, que desde 1834 funcionava no edifício antes ocupado pelos Terceiros (Campos, 2013; Anselmo, 2014). Acrescente-se que na biblioteca da Faculdade de Letras das Universidade de Lisboa se encontra um exemplar da *Geografia* datado de 1520, equivalente àquele presente na BNP e que em 1776 pertencia a um “Luiz Coelho Amado”, possível parente do mestre impressor setecentista Manuel Coelho Amado. Apesar de desconhecermos a proveniência do exemplar da Faculdade de Letras, recorda-se que essa faculdade funcionou durante um século no edifício partilhado com a Academia das Ciências, no Bairro Alto, antes da sua transferência para a atual localização na Cidade Universitária, em 1958.

A biblioteca do palácio da Ajuda tem um exemplar da edição romana de 1490 e outro da edição de Ingolstadt, de 1533. O primeiro ostenta a marca de posse da Biblioteca das Necessidades, que em 1834 absorveu a biblioteca da então extinta Casa de Nossa Senhora das Necessidades, da Congregação do Oratório – a mesma congregação religiosa que detivera o outro exemplar do mesmo incunábulo de 1490 que vimos ter transitado para a Biblioteca Nacional. Em 1910, na sequência da implantação da República, a biblioteca do Palácio Real das Necessidades foi integrada no Palácio da Ajuda (Campos, 2013). Por seu turno, na biblioteca do antigo Convento de Mafra está um exemplar da edição veneziana da *Geografia* de Ptolomeu de 1574, assim como o referido exemplar da *Historiae Britanicae, Saxonicae, Anglo-Danicae scriptores XV*, de Thomas Gale, de 1691. Construída por impulso de dom João V, essa biblioteca servia sobretudo os frades e os estudantes que frequentavam a escola aí instalada (Cabral, 2014b).

Integram ainda a lista dos originais da *Geografia* sobreviventes nas bibliotecas de Lisboa os exemplares das edições venezianas de 1561 e 1598 existentes na Biblioteca Central de Marinha. Este último exemplar indica pertença de “J. Biker e Joannis Attily à Salzburg”, seguida da do comandante Álvaro Augusto Nunes Ribeiro (1878-1933), que legou à Biblioteca Central de Marinha um espólio constituído por 675 volumes, incluindo numerosos exemplares de livro antigo dos séculos XVI a XVIII (Valentim, 2003). Na Biblioteca Universitária João Paulo II, da Universidade Católica Portuguesa, estão outros dois espécimes da *Geografia* de Ptolomeu: a edição de Veneza de 1598 e a edição de Pádua de 1621.

Na Sociedade de Geografia de Lisboa encontra-se um exemplar da edição veneziana de 1574 da qual também há exemplares equivalentes nos fundos da BNP, da Biblioteca Geral da Universidade de Coimbra, da Biblioteca Pública Municipal do Porto e da Biblioteca do Palácio de Mafra, como referimos. Essa edição e a de 1525, de Estrasburgo, revelam ser as mais repetidas no conjunto das bibliotecas portuguesas que rastreámos. O rastreio fica completo com a notícia da existência, na Biblioteca Pública de Évora, de um único exemplar da edição veneziana de 1562.

## Elementos para uma síntese: circulação e públicos de um livro científico

Se a ciência moderna se funda no estudo matemático da realidade material, os argumentos quantitativos que sustentam a *Geografia* de Ptolomeu, correspondentes a um inventário do mundo com base na apresentação da posição precisa dos lugares segundo coordenadas de latitude e longitude, fizeram dessa obra o protótipo de “livro científico” (Leitão, 2004; Jacob, 2008). A sua redescoberta pelo Renascimento italiano no dealbar do século XV, junto com o poderoso efeito propulsor dado pelo desenvolvimento da imprensa, trouxe consigo uma dupla consequência: um interesse renovado pela cosmografia, ou seja, por esse campo do saber com raízes clássicas e medievais que estuda o mundo no seu conjunto, em paralelo com uma atenção mais circunscrita à descrição da ecúmena que Ptolomeu tratara como “geografia”. A ambiguidade terminológica que acompanhou durante muito tempo as modernas traduções desse livro, hesitando, precisamente, entre chamar-lhe *Cosmografia* e *Geografia*, apenas reforça a sua importância nesse processo pelo qual a cosmografia assume um lugar da maior relevância tanto na história da ciência como na história da geografia (Clare, 2023).

É sabido que uma comunidade de agentes, saberes e redes de comunicação muito diversas, umas mais instituídas, outras mais “invisíveis”, contribuiu para a disseminação dos principais conteúdos inscritos na *Geografia* de Ptolomeu através da Europa. Como seria de esperar, também em Portugal identificámos audiências e leitores muito diferentes desse livro científico: dos espaços dos conventos e outras instituições religiosas aos lugares de ensino universitário, dos círculos cortesãos e das elites comerciais a agentes mais inesperados ou heterodoxos. Para o nosso exercício assumimos que a biblioteca constitui o lugar de mediação decisivo entre sucessivas comunidades de leitores. Mesmo antes de se instituir a moderna compartimentação e autonomia dos saberes disciplinares, o estudo, ainda que circunscrito, das coleções e objetos custodiados nesses espaços, oferece uma janela privilegiada para reconstituirmos a história da produção, circulação e leitura de uma obra científica como a que nos ocupou (Pombo, 1997; Leitão, 2004).

Exceção feita à tradução muito parcial devida a Pedro Nunes no seu *Tratado da esfera*, de 1537, os prelos portugueses não produziram nenhuma edição própria da *Geografia* de Cláudio Ptolomeu até 1730, limite máximo compreendido pelo nosso estudo. Mas isso não obstou a que os principais conceitos geográficos, matemáticos e cartográficos introduzidos nessa obra tenham sido cedo assimilados, difundidos e discutidos em Portugal, desenhando um panorama de receção alinhado com a restante Península Ibérica.

O número expressivo de exemplares da *Geografia* de Ptolomeu que localizámos nas principais bibliotecas portuguesas – assim como a presença da maioria das edições impressas pelos prelos alemães, italianos e franceses que se encarregaram da difusão europeia deste livro ao longo dos dois séculos seguintes ao da sua redescoberta pelo Ocidente – atesta uma atenção reiterada pelos conteúdos de geografia matemática e pela ideia do mundo legados por Ptolomeu. A presença desse livro em boa parte das bibliotecas onde foi custodiado era acompanhada por número significativo de obras de autores clássicos gregos e latinos, assim como de autores da antiguidade cristã, sendo os mais representativos Flávio Josefo, Heródoto, Tucídides, Tito Lívio, Políbio, Eusébio de Cesareia e são Jerónimo (Campos, 2013). Indiscutivelmente, foi nesses meios eruditos, correspondentes ao universo dos conventos e casas monásticas, tal como aos círculos da Corte, que detetámos o traço dos principais agentes cultivados em saber teológico, canonístico e filosófico, a quem esse livro mais interessou. Cabem também neste perfil as apropriações tardias de alguns dos seus conteúdos que notámos terem acontecido em geografias de Portugal setecentistas de perfil erudito, como as que foram escritas pelo padre João Baptista de Castro.

Paralelamente, ocorrem exemplos da posse desse livro por parte de comerciantes estrangeiros residentes em Lisboa, assim como de diplomatas portugueses com extensa carreira nas capitais europeias, anunciando o perfil do bibliófilo ilustrado que fará desse objeto uma peça importante das suas coleções de livros raros e antigos. A progressiva modificação dos significados científico e cultural atribuídos ao livro de Ptolomeu é acelerada pelo processo de dispersão dos antigos fundos conventuais pelas bibliotecas públicas e administrativas portuguesas que decorre do triunfo do liberalismo, em 1834, concomitante a instituição das grandes bibliotecas públicas e de Estado consolidadas no século XIX europeu. Reiteramos: antever toda essa sucessiva modificação de usos e sentidos a partir do caso singular dado pela *Geografia* permite realizar uma aproximação pertinente a algumas das questões centrais da circulação do livro antigo científico num contexto nacional bem definido como o português.

## Data Availability

Não estão em repositório.
